# Effect of traditional Chinese medicine formula Sinisan on chronic restraint stress-induced nonalcoholic fatty liver disease: a rat study

**DOI:** 10.1186/s12906-017-1707-2

**Published:** 2017-04-07

**Authors:** Fafeng Cheng, Chongyang Ma, Xueqian Wang, Changming Zhai, Guoli Wang, Xiaolin Xu, Jie Mu, Changxiang Li, Zisong Wang, Xiaoyu Zhang, Wenchao Yue, Xin Du, Yajun Lian, Wenxiang Zhu, Xiangjun Yin, Zhen Wei, Wenjie Song, Qingguo Wang

**Affiliations:** School of Basic Medical Sciences, Beijing University of Chinese Medicine, 11 Beisanhuandong Road, Chao yang District, Beijing, 100029 China

**Keywords:** Psychological stress, Chronic stress, NAFLD, TCM, Sinisan, Oxidative stress

## Abstract

**Background:**

Nonalcoholic fatty liver disease (NAFLD) represents one of the most common forms of liver disease worldwide, and it is always regarded as a consequence of a sedentary, food-abundant lifestyle, sitting for an extended time, and a low physical activity level, which often coincide with chronic and long-lasting psychological stress. A Chinese medicine Sinisan (SNS) may be a potential formula for treating this kind of disease.

**Methods:**

In this study, a long-term chronic restraint stress protocol was used to investigate the mechanism underlying stress-induced NALFD. To investigate the effect of SNS treatment on stress-induced NAFLD, we measured the liver and serum values of total cholesterol (TC), triglyceride (TG), liver free fatty acids (FFA), low-density lipoprotein, superoxide dismutase, tumor necrosis factor-α, malondialdehyde, interleukin (IL)-6, and serum values of aspartate aminotransferase (AST), alanine aminotransferase (ALT), and alkaline phosphatase. Results are shown as a mean ± standard deviation. Significant differences between the groups were evaluated using the Student t-test. For multiple comparisons, one-way analysis of variance (ANOVA) was used. If the results of ANOVA indicated significant differences, post hoc analysis was performed with the Tukey test or Dunnett test, and *p* < 0.05 was considered statistically significant.

**Results:**

Long-term chronic stress led to steatosis and non-alcoholic steatohepatitis. Additionally, SNS treatment significantly increased body weight gain (*p* < 0.01) and sucrose preference (*p* < 0.001), and it reduced the liver values of TC, TG, and FFA (*p* < 0.05). SNS also reduced the serum values of AST and ALT (*p* < 0.001), and the liver value of IL-6 (*p* < 0.01).

**Conclusions:**

This study’s results demonstrate that psychological stress may be a significant risk factor of NAFLD. Furthermore, the traditional Chinese medicine formula SNS may have some beneficial effect in antagonizing psychological stress and stress-related NAFLD.

## Background

Nonalcoholic fatty liver disease (NAFLD) represents one of the most common forms of liver disease worldwide [1], and is considered a hepatic manifestation of the metabolic syndrome [2]. NAFLD refers to a wide spectrum of liver injuries, ranging from simple fat accumulation (steatosis) to other severe liver diseases such as non-alcoholic steatohepatitis (NASH), fibrosis, cirrhosis, and hepatocellular carcinoma [3]. The prevalence of NAFLD has increased remarkably over the last three decades to nearly 15–30% in the general population in western countries [4]. Recent data on epidemiology of NAFLD, suggest that this is the most common liver disease worldwide. Not only in western Countries, but also in India, China and sud America [5]. In China, its prevalence approximately doubled in the past decade and reached 15–20% in affluent regions [6, 7]. The mechanism involved in the development of NAFLD is still under debate, although several hypotheses have been proposed. One generally accepted theory is the two-hit hypothesis [8], which suggests that the accumulation of fat in the liver predisposes the liver to secondary stress such as oxidative stress, inflammation, and cytokines [9]. Actually, no treatment for NAFLD was codified nowadays. The international guidelines report that lifestyle changes are the only therapies suggested in NAFLD patients, in particular in overweight/obese [10]. However, many complementary therapies and in particular herbal medicine, are been studied to treat NAFLD.

The prevalence of NAFLD is always regarded as a consequence of a sedentary, food-abundant lifestyle, sitting for a long time, and low physical activity level [11, 12], which often coincide with long-lasting and chronic stress. Stress is defined as any physical or psychological change that could interfere with one’s internal environment [13], thereby increasing the risk of various health problems. It is also characterized by the activation of the autonomic nervous system, hypothalamic-pituitary-adrenal axis, and sympathetic-adrenal-medullary system [14]. In modern society, which is characterized by a rapid pace of life, individuals ceaselessly encounter an increasing number of psychological stressful stimuli such as emotional stimuli and social stress [15]. It had been validated by clinical trials that chronic stress from high levels of working pressure and a low quality of life are the risk factors of obesity and metabolism syndrome [16, 17]. Furthermore, chronic stress can lead to multiple systemic inflammation, hypoxic organs, and oxidative stress [18–20]. Thus, it is likely that chronic stress directly and indirectly promotes the progression of chronic liver disease. Although some studies have shown that long-term and repeated chronic stress affect high-fat diet-induced NAFLD progression [21], the relationship between chronic stress and NAFLD is still unclear. Therefore, we are eager to determine whether chronic stress can lead to NAFLD. Further exploration into the mechanism of stress-induced NAFLD will contribute to the discovery of novel therapeutic targets and the design of optimized treatment strategies for patients with NAFLD.

Sinisan (SNS), also called Shigyaku-san in Japan, originated from the book of Shang Han Lun written during the Han Dynasty (200–201 AD) by a famous Chinese doctor Zhongjing Zhang, and it comprises four herbal medicines: Aurantii Immaturus Fructus (Zhi-Shi), Paeonae Alba Radix (Bai-Shao), Licorice Root (Gan-Cao), and Bupleuri Radix (Chai-Hu) with a dose proportion of 1:1:1:1 [22]. Some studies have demonstrated its effectiveness against liver injury [23, 24], hepatitis [25, 26] and Palmoplantar hidrosis [27]. Actually, SNS is proposed to reverse impairments in spatial learning and memory, reduces aggressive behavior in rats subjected to chronic restraint stress [28], and SNS alone was also more effective in reducing depression-like behavior than fluoxetine alone, and markedly increased central 5-HT and reduced peripheral 5-HT in model rats [29, 30]. Although there is no proven therapy for NAFLD, an experimental study indicated that SNS could prevent the course of NASH by adjusting TG distribution in serum and liver, increasing the anti-oxidative capacity of liver, and reducing the peroxide damage in a MDC-diet NAFLD model [31]. Otherwise, some components of SNS, such as paeoniflorin [32] and glycyrrhetic acid [33], are reported to have protective effect against NAFLD in vivo and in vitro NAFLD models. Taken together, we noticed that SNS may have potential effect on stress-induced depression, hepatic steatosis and steatohepatitis. Therefore, the purposes of the present study were to compile evidence for this stress-induced NAFLD hypothesis using the chronic restraint stress (CRS) model in rats, and to investigate potential therapeutic effects of SNS for stress-related NAFLD as an alternative and safe drug for clinical use.

## Methods

### Preparation of SNS extract

Crude preparations of SP were purchased from Beijing Tongrentang (Beijing, China). The herbs were authenticated by our team. The SNS extract was prepared according to the previous article [34]. This extract contained flavonoid glycosides (56.36%) and saponins (12.067%), which were detected by UV. Neohesperidin (24.547%), naringin (20.2%) and glycyrrhizic acid (2.654%) were measured by high performance liquid chromatography coupled to UV detection, which were consistent with the article.

### Experimental animals and treatments

In the present study, 30 male specific-pathogen-free level Sprague-Dawley rats (weighing 130–150 g) were commercially obtained from Vital River Laboratories (number SCXK 2012–0001), and they were permitted to acclimate to the environment for 7 days before the experiments began. Then they were randomly assigned to the control, stress, and SNS treatment groups (*n* = 10, respectively). Control rats were left undistributed, whereas rats in the stress and SNS groups had chronic stress for 9 weeks. Details of this process are described in Stress Protocol section. Rats in the control group and stress group were gavaged with sterile water at a dose of 10 mL × kg^−1^ of body weight. Rats in the SNS group received SNS at a dose of 3.6 g × kg^−1^ of body weight by gavage. All animals were treated daily from 8:30–9:00 am. The treatments lasted for the entire experiment. The animals were maintained under a standard 12 h light/dark cycle (lights on at 8:00 a.m.) at a constant temperature of 24 ± 1 °C and relative humidity of 45 ± 15% with free access to food and distilled water, except for periods of water and food deprivation.

### Stress protocol

**Fig. 1:**
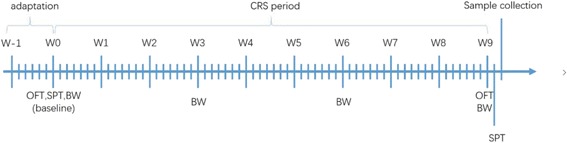
Schedule of the procedures used in the present study. The tests included the open-field test (OFT) and sucrose preference test (SPT). CRS, chronic restraint stress; BW, body weight

The long-term chronic restraint stress procedure was performed according to a previous study [35] with minor modifications. This procedure mimics stress that is largely psychological in nature. In brief, restraint stress was applied using a plastic restrainer (550-mL cubage water bottle, Nongfu Spring Company Limited and 600-mL cubage water bottle, Danone) with multiple punctures that enabled it to closely fit over the mice. The rats were restrained for 6 h per day for 9 consecutive weeks. The stress procedure was conducted at the institutional animal facility throughout the experimental period between 10 am and 4 pm. Immediately after terminating the stress exposure, animals returned to their home cages. The control rats were not disturbed during the 9-week period, and they remained isolated from the stressed animals with access to food or water during the same period of stress to avoid any acoustic or olfactory communication between the groups. The time schedule for the CRS procedure is shown in Fig. 1.

### Experimental procedures

After 9 weeks, all rats were fasted overnight and then anesthetized using isoflurane. Blood was collected by orbital puncture, followed by sacrifice through cervical dislocation. Next, the entire liver was resected. For histology, a small part of the liver was immediately fixed in 10% formalin and processed for paraffin embedding. The rest of the liver was stored at −80 °C for further biochemical analysis.

### Open-field test

The open-field test (OFT), performed as previously described, was used to study the exploratory and anxious behavior of the rats [36]. The open-field test was conducted between 8:00 am and 12:00 am in a quiet room (≤60 dB) pre-dosing. The open-field apparatus consisted of a black square arena (100 cm × 100 cm) with a black wall (40 cm high). The floor was marked with a grid that divided the floor into 25 equal-size squares. During a 3-min observation period, each rat was placed at the center of the apparatus, and horizontal locomotion (the number of total squares crossed) and the frequencies of rearing (defined when the rat stands upright on its hind legs) were recorded. Each animal was tested using the apparatus once. Simultaneously, the body weight of each rat was recorded.

### Sucrose preference test

The sucrose preference test was based on a previous study [37]. Seventy-two hours before the test, rats were trained with 1% sucrose solution (*w*/*v*); two bottles of 1% sucrose solution were placed in each cage, and 24 h later, 1% sucrose in one bottle was replaced with tap water for 24 h. After adaptation, the rats were deprived of water and food for 24 h, followed by a sucrose preference test, in which each rat was placed in an individual metabolic cage that contained two bottles, one with water and one with 1% sucrose solution. The test lasted for 1 h. Sucrose preference was defined as [sucrose intake/(sucrose intake + water intake)] × 100%.

Hematoxylin-eosin and oil red O staining of the liver.

Following fixation of the livers with a 10% formalin/phosphate-buffered saline, the livers were sliced and stained with hematoxylin-eosin (HE) for histological examination. According to the NASH Clinical Research Network Scoring System [38], liver steatosis was graded semi-quantitatively based on the percentage of hepatocytes according to the following criteria: grade 0, <5% of hepatocytes involved; grade 1, 5–33% of hepatocytes involved; grade 2, 34–66% of hepatocytes involved; and grade 3, >66% of hepatocytes involved. Liver inflammation was graded based on an overall assessment of all inflammatory foci according to the following criteria: grade 0, no foci; grade 1, <2 foci per ×200 field; grade 2, 2–4 foci per ×200 field; and grade 3, >4 foci per ×200 field. Hepatic lipid content was also determined by staining with oil Red O (Sigma-Aldrich).

### Serum biochemical analysis

The serum triglyceride (TG), total cholesterol (TC), alanine aminotransferase (ALT), and aspartate aminotransferase (AST) levels were measured using an automatic biochemistry analyzer (CX4/Pro, Beckman Coulter).

### Measurement of the hepatic lipids

The livers were homogenized at 4 °C in a lysis buffer containing 50 mmol/L Tris (pH 8.0), 150 mmol/L NaCl, 1% Triton X-100, and 0.5% sodium deoxycholate. Lipids from the total liver homogenate were extracted using the chloroform/methanol method (2:1), evaporated, and dissolved in 2-propanol.

### Measurement of low-density lipoprotein, superoxide dismutase, malondialdehyde, and cytokines

The liver low-density lipoprotein (LDL) level was estimated using commercially available kits (Jian Cheng Biological Engineering Institute). A Superoxide Dismutase Detection Kit (Jian Cheng Biological Engineering Institute) was selected to measure superoxide dismutase (SOD) levels. A Malondialdehyde (MDA) Detection Kit (Jian Cheng Biological Engineering Institute) was selected to determine the MDA level as a marker of lipid peroxidation. The liver tumor necrosis factor (TNF)-α and interleukin (IL)-6 levels were determined using an enzyme-linked immunosorbent assay kit (Cloud-Clone Corp.). All assays were performed according to the manufacturers’ instructions.

### Data analysis

Results are shown as a mean ± standard deviation (SD). Statistical analysis was performed using SPSS 23.0 software (SPSS, Inc.). Significant differences between the groups were evaluated using the Student t-test. For multiple comparisons, one-way analysis of variance (ANOVA) was used. If the results of ANOVA indicated significant differences, post hoc analysis was performed with the Tukey test or Dunnett test, and *p* < 0.05 was considered statistically significant.

## Results

### Effect of SNS on chronic stress-induced body weight and stress-related behavior

**Fig. 2:**
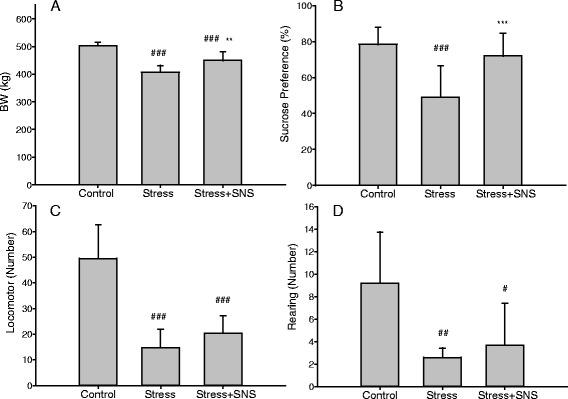
Changes in behavioral indicators after the chronic restraint stress (CRS) procedure and Sinisan (SNS) treatment. **a** Body weight (BW) changes after CRS. **b** The sucrose preference after the CRS procedure and SNS treatment. **c**, **d** Open-field test indexes (the number of locomotor and rearing events) after the CRS procedure and SNS treatment. Values plotted are mean ± standard deviation (*N* = 10 per group). #*p* < 0.05, ##*p* < 0.01, ### *p* < 0.001 versus the control group, ** *p* < 0.01, *** *p* < 0.001 versus the stress group

At the end of the experimental protocol, body weight gain was significantly lower in the stress group than in the control group (*p* < 0.001), and the SNS group had a higher body weight gain than the stress group (*p* < 0.01) (Fig. 2a). The sucrose preference was lower in the stress group than in the control group (*p* < 0.001), and the sucrose preference was higher in the SNS group than in the stress group (*p* < 0.001) (Fig. 2b). The numbers of locomotor (*p* < 0.001) and rearing (*p* < 0.01) events in the OFT were significantly lower in the stress group than in the control group; however, there was no significant difference between the stress group and SNS group (Fig. 2c, d).

Relationship between chronic stress and steatosis, and NASH.

**Fig. 3:**
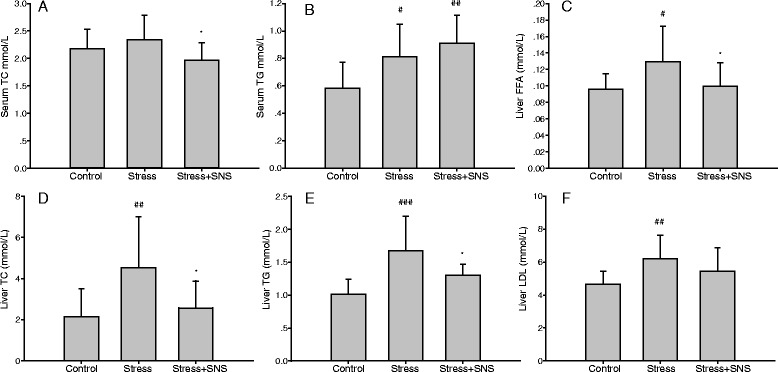
Effects of chronic stress. Chronic stress induces the accumulation of hepatic triglyceride (TG), total cholesterol (TC), free fatty acids (FFA), and low-density lipoprotein (LDL), and Sinisan (SNS) treatment can decrease this accumulation. **a** The serum TC level is shown in the bar chart. **b** The serum TG level is shown in the bar chart. **c** The liver FFA level. **d**, **e** The liver TG and TC concentrations in each group. **f** The liver LDL level. Values plotted are a mean ± standard deviation (*N* = 10 per group). #*p* < 0.05, ##*p* < 0.01, ### *p* < 0.001 versus the control group, * *p* < 0.05 versus the stress group

The serum TC level had an increasing tendency but was not statistically significant, and the serum TG level significantly increased (*p* < 0.05) (Fig. 3a, b). Results of quantitative analysis showed a significant increase in the liver values of FFA (*p* < 0.05), TG (*p* < 0.01), TC (*p* < 0.001), and LDL (*p* < 0.01) in stressed rats (Fig. 3c-f). Therefore, these data suggest that chronic stress can induce liver steatosis independent of the dietary factor.

**Fig. 4:**
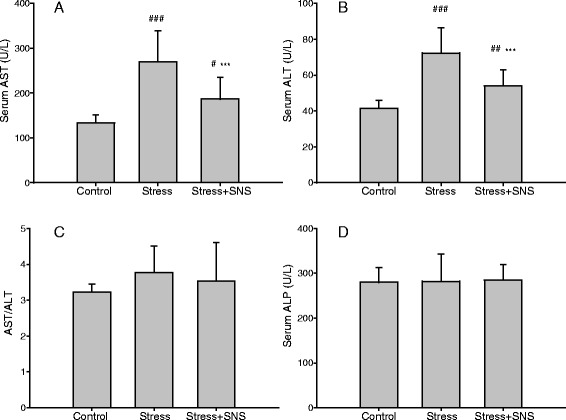
The serum activities of three hepatic injury-associated enzymes, alanine aminotransferase, aspartate aminotransferase, and alkaline phosphatase. **a** The serum AST level. **b** The serum ALT level. **c** The AST and ALT ratio. **d** The serum ALP level. Values plotted are a mean ± standard deviation (*N* = 10 per group). ###*p* < 0.001 versus the control group, *** *p* < 0.001 versus the stress group. SNS, Sinisan; ALT, alanine aminotransferase; AST, aspartate aminotransferase; ALP, alkaline phosphatase

**Fig. 5:**
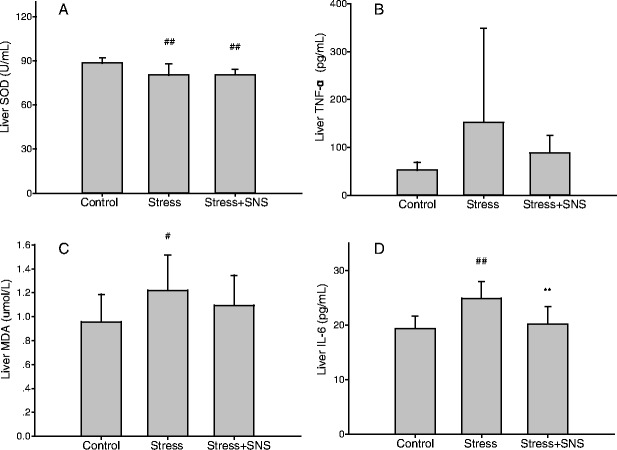
The oxidative stress and inflammation of the liver. **a** The superoxide dismutase (SOD) level of each group. **b** The tumor necrosis factor (TNF)-α level of each group. **c** The malondialdehyde (MDA) level of each group. **d** The liver interleukin (IL)-6 level of each group. Values plotted are a mean ± standard deviation (*N* = 10 per group). #*p* < 0.05, ##*p* < 0.01 versus the control group, **p < 0.01 versus the stress group. SNS, Sinisan

Results showed that stressed rats developed exacerbated NASH compared to the control rats, as indicated by increased levels of serum AST (*p* < 0.001) and ALT (*p* < 0.001), and NAFLD activity inflammation scores (*p* < 0.01) (Fig. 4a, b). No significant difference was observed in the serum alkaline phosphatase (ALP) level and AST/ALT ratio between the stress and control groups (Fig. 4c, d). Regarding the SOD (*p* < 0.01), MDA (*p* < 0.05), and IL-6 (*p* < 0.01) levels, stressed rats had higher liver levels than control rats, and concerning the TNF-α level, no significant increase was observed in the stress group (Fig. 5a-d).

### Effect of SNS on hepatic steatosis and liver injury

**Fig. 6:**
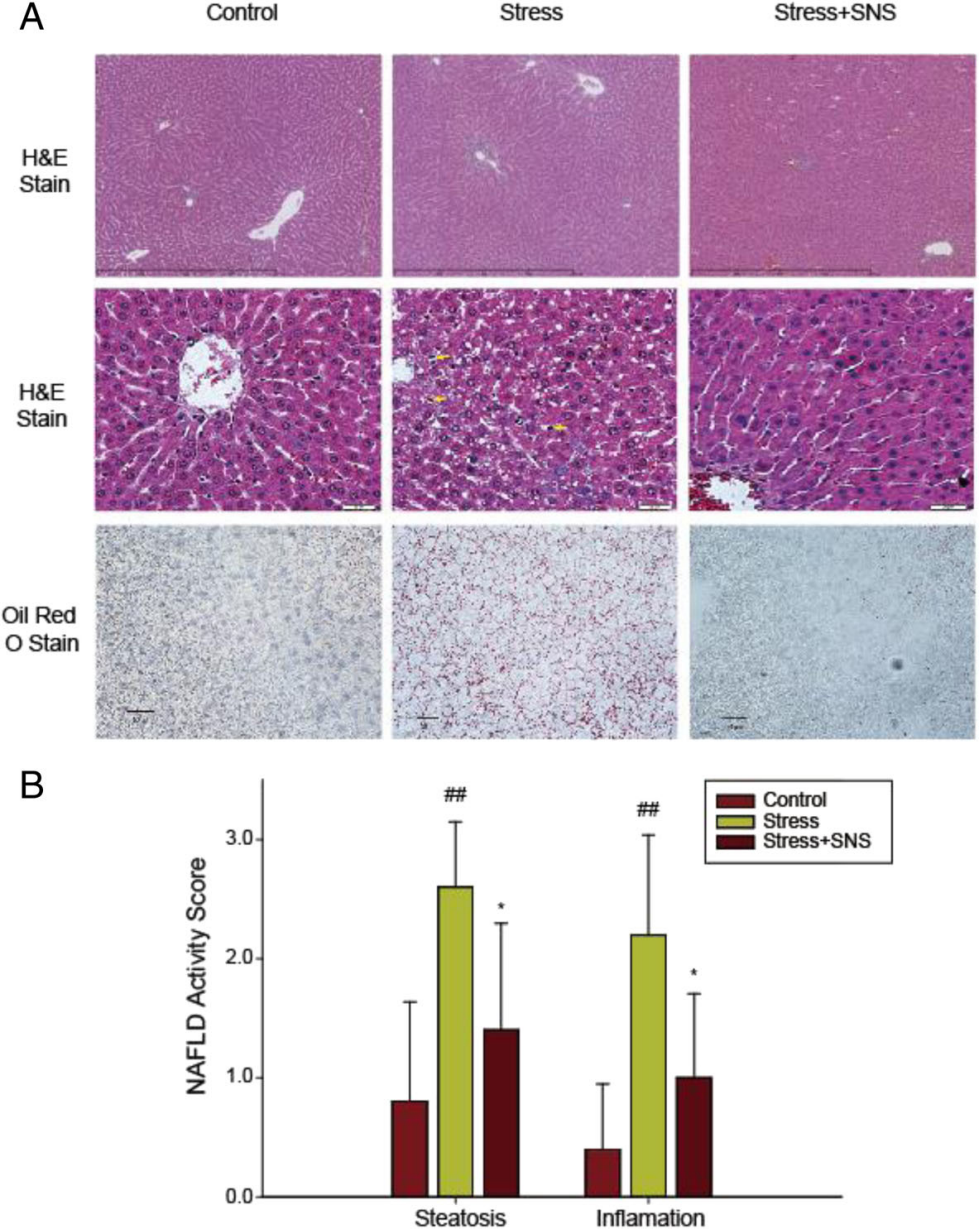
Photomicrographs of histological changes in the rat liver. **a** Representative slides show hematoxylin and eosin (HE)-stained and oil red O-stained liver sections from rats of each group. HE sections show chronic stress causing foci of inflammatory cell infiltration and lipid deposits (yellow arrows) (original magnification, ×100 and ×400, respectively). **b** The nonalcoholic fatty liver disease (NAFLD) activity score was evaluated using the New York NAFLD score system. Values plotted are a mean ± standard deviation (*N* = 10 per group). ##*p* < 0.01 versus the control group, *p < 0.05 versus the stress group

In vivo, our results showed that after 9 weeks of restrained stress, SNS treatment prevented increases in the levels of serum TC, liver FFA (*p* < 0.05), and liver TC (*p* < 0.05) and TG (*p* < 0.05) in the chronic stress condition (Fig. 3c-e). Additionally, no significant change was observed in the serum TG and liver LDL levels between the stress and SNS groups (Fig. 3a, f). The serum TG level had an increasing tendency in the SNS group compared to the stress group. Compared to the stress group, the levels of serum AST (*p* < 0.001) and ALT (*p* < 0.001) in the SNS group were significantly decreased (Fig.4 a, b), indicating that SNS may have the ability to ameliorate NAFLD-related liver injury. No difference in the serum ALP level was observed among the groups (Fig. 4d). Chronic stress was confirmed to a certain degree by histological staining using oil red O (Fig. 6a). Lastly, according to the pathological analysis, the liver steatosis and inflammation score indicated that SNS treatment (*p* < 0.05) can ameliorate hepatic steatosis and NASH (Fig. 6b).

### Effect of SNS on hepatic inflammation and oxidative stress

Lipid accumulation may link oxidative stress to inflammation, forming a feedback loop that significantly aggravates the severity of NAFLD [39]. As shown in Fig. 5d, compared to the stress group, the SNS group exhibited decreased levels of pro-inflammatory cytokine IL-6 (*p* < 0.01). No significant difference was observed in the levels of SOD, MDA, and TNF-α (Fig. 5a-c), but all these makers had a normalizing trend.

## Discussion

Over the past decade, NAFLD has become one of the most prevalent causes of chronic liver disease [40]. Accumulating evidence has shown that psychological stress may correlate with the increasing incidence of metabolic syndrome, inflammation, and NAFLD [41, 42]. However, previous studies mostly concentrated on the effect of chronic stress on obesity or metabolic syndrome combined with a high-fat diet [43, 44]. In the present study, the key finding was that rats could develop steatosis and NASH only during the intervention of chronic stress, although their body weight gain was decreased. Furthermore, chronic stress exposure can lead to oxidative stress and a chronic inflammatory state in the liver. To exclude the dietary factor and other related factors in this experiment, we chose a mimics restraint stress model that is largely psychological in nature.

In the current study, the pathological results showed that hepatic lobule structures were disorderly arranged in stressed rats. Hepatic steatosis and inflammatory cell infiltration were also observed. The levels of serum TG and liver FFA, TC, TG and LDL were significantly increased, suggesting the existence of hepatic fat deposition. Particularly, the increased retention of lipids in hepatocytes, mostly in the form of TG and TC, is the common early trait of NAFLD. Additionally, we found that chronic stress can lead to a high level of serum ALT and AST, indicating a capacity of chronic stress for hepatocyte injury. A high level of liver IL-6 and MDA, and low level of liver SOD were also found. IL-6 is a pro-inflammatory pleiotropic cytokine produced by adipocytes, hepatocytes, and immune and endothelial cells [45]. Some studies have shown that accumulated FFAs in hepatocytes activate IκB kinase and nuclear factor-κB, a transcription factor that plays a central role in coordinating the expression of various pro-inflammatory cytokines, including IL-6 [46]. Chronic exposure to IL-6 led to liver injury, although studies have concluded that it has a protective role against the progression of hepatic steatosis, and paradoxically, a hepatocytes protective role in advanced stages of NAFLD [47, 48]. Additionally, MDA and SOD are important markers of oxidative stress. However, MDA is a kind of aldehyde produced through the lipid peroxidation process, which can promote crosslinking of nucleic acids, proteins, and phospholipids and change the function of these biological macromolecules. The level of lipid peroxidation can be represented by MDA content [49, 50]. Conversely, SOD can transform superoxide anions into H_2_O_2_, thereby eliminating superoxide anions and protecting cells from oxygen radical injury. Therefore, SOD is the main factor that protects the liver tissue against harmful oxidative stress from internal and external environments [51]. An increased MDA level and decreased SOD activity are important signs of increased oxidative stress [52, 53]. Therefore, these results further confirmed that the second hit was involved in the pathogenesis of stress-related NAFLD.

In terms of TCM, qi is an active principle forming part of any living thing. The dredging function of the liver is extremely important in regulating the flow of Qi and blood in the body. It is one of the most vulnerable organs to anger, repression, and distress resulting in liver qi stagnation, which produces typical symptoms of stress-related disorders. We believe that the theory of liver qi has some relationship with psychological stress. In the present study, we found that psychological stress can lead to NAFLD and even NASH, which means that the liver may be an organ that stress targets, thus proving the liver qi theory to some extent. In China, SNS has a nearly 2000-year history of use for treating patients with liver qi stagnation, and it has shown some curative effects. Some studies have shown that SNS is effective for improving disorders of the digestive system, including liver injury [54] and colitis [55]. Theoretically, SNS may be the most appropriate herb formula for treating stress-related NAFLD. Therefore, we investigated the mechanism of SNS for treating NAFLD. Our results showed that SNS can increase the sucrose preference of stressed rats, which means that SNS may decrease anhedonia-like behavior, which is the main symptom of depression in humans [56]. No significant difference was observed in OFT, indicating that SNS may have little effect on the activity and excitability of stressed rats. Therefore, SNS may be a useful anti-stress formula, which is in agreement with findings from another study on rats [28]. Our data also showed that SNS treatment can significantly improve hepatic steatosis and reduce the AST and ALT levels. Moreover, the serum TC level and liver FFA, TC, and TG levels of the SNS treatment group were significantly decreased. Similarly, we found that SNS treatment decreased the level of liver IL-6, which suggests that the beneficial effect of SNS on NAFLD may be partly attributable to the anti-inflammatory effect, including the down-regulated expression of IL-6. SNS can increase and reduce the SOD and MDA levels, but this trend was not statistically significant, suggesting that it is uncertain whether SNS improve NAFLD by reducing oxidative stress. Overall, the beneficial effect of SNS involves anti-steatosis and anti-inflammation.

## Conclusions

Our study provides new insight into the TCM liver qi theory and the pathogenesis of NALFD. Our findings strongly support that psychological stress may be a significant risk factor of NAFLD, although our experiment could not completely eliminate physical agents. Furthermore, TCM formula SNS may have some beneficial effect in antagonizing psychological stress and stress-related NAFLD. These results serve to remind physicians that those with long-term psychological stress may also be at risk for developing NAFLD, regardless of their weight. To prevent NAFLD, physicians should concentrate on stress management and clinically apply the TCM theory and formula SNS in patients with psychological stress and NAFLD.

## Abbreviations

ALP: Alkaline phosphatase
ALT: Alanine aminotransferase
ANOVA: Analysis of variance
AST: Aspartate aminotransferase
CRS: Chronic restraint stress
FFA: Free fatty acids
HE: Hematoxylin-eosin
IL: Interleukin
LDL: Low-density lipoprotein
MDA: Malondialdehyde
NAFLD: Nonalcoholic fatty liver disease
NASH: Non-alcoholic steatohepatitis
SD: Standard deviation
SNS: Sinisan
SOD: Superoxide dismutase
TC: Total cholesterol
TCM: Traditional Chinese medicine
TG: Triglyceride
TNF-α: Tumor necrosis factor-α
